# How to create a mindful community of practice: exploring the social functions of group-based mindfulness practices facilitated via Zoom during COVID-19

**DOI:** 10.3389/fpsyg.2025.1356057

**Published:** 2025-01-29

**Authors:** Jutta M. Tobias Mortlock, Hotri Himasri Alapati, Trudi Edginton

**Affiliations:** Department of Psychology, School of Health and Psychological Sciences, City St George’s, University of London, London, United Kingdom

**Keywords:** mindfulness, online, COVID-19, community of practice, interbeing, social connection, interdependence theory, meditation

## Abstract

This exploratory qualitative study was conducted to investigate the experiences of individuals who have been participating in online mindfulness sessions with an online mindfulness community since the beginning of COVID-19, i.e., during a period of heightened uncertainty and social isolation. The study’s purpose was to better understand the social functions of regularly practicing mindfulness in this online community of practice. Analyses from semi-structured interviews reveal how shared mindfulness practice may foster several pillars of connection and interbeing in this community of practice. These include improved mind–body awareness, coupled with a unique sense of trust and connection, which may have helped cultivate collective alignment and a sense of common humanity among research participants. Findings are discussed through the lens of interdependence theory, resulting in several exploratory propositions on how to create a mindful community of practice. The study concludes with a call for more research in this understudied research domain and invites mindfulness researchers and practitioners to test these propositions further. Its overall aim is to stimulate debate among individuals and groups intent on creating a mindful community in their workplace, educational setting, or neighborhood.

## 1 Introduction

In Eastern contemplative traditions, mindfulness is considered a method – or practice – with a specific purpose: to develop lucid, metacognitive awareness of one’s experience in order to clearly comprehend and transform suffering (Bodhi, 2011). In the scientific literature, the link between mindfulness and well-being has been extensively studied and mindfulness meditation is now widely utilized as part of mental health interventions (Wielgosz et al., 2019) including in workplaces (Kelloway et al., 2023). Furthermore, leading mindfulness scholar Jon Kabat-Zinn argues that mindfulness has transformative potential: mindfulness helps cultivate capacity to alleviate suffering and promote wellbeing for individuals as well as for communities and the world at large (Kabat-Zinn, 2011). In this paper, we incorporate Kabat-Zinn’s assertion about the transformative potential of mindfulness and lean on Bodhi (2011) and Kudesia (2019) to define mindfulness by its purpose, as a metacognitive practice to deeply understand and transform suffering and generate wellbeing, for one and all.

Communities of practice are groups of individuals who come together regularly to learn together, to share knowledge, and to benefit from belonging to a community of shared interests (Wenger, 1998). When people practice mindfulness together regularly, they can be considered a mindful community of practice. Typically these initiatives last two to three months, and then the intervention stops. But what happens when people in a workplace come together for a longer period of time to practice mindfulness? What are the social functions (in other words, the beneficial effects of actions or processes in a social system; Merton, 1949) of group-based mindfulness practice in an online mindfulness community of practice created during COVID-19? This is the question at the heart of our study.

In this paper, we focus our attention on the transformative potential of mindfulness. Specifically, our work responds to calls for more research on how mindfulness may help generate wisdom and transform suffering, not only for individuals but for everyone (Bahl et al., 2016; Daniel et al., 2022; Tobias Mortlock, 2023). Scholars have theorized on why and how mindfulness can be transformative beyond beneficial individual change, for social groups and even for society as a whole. For example, du Plessis and Just (2022) argue that mindfulness can transform the way we think about ourselves and others through critical reflexivity. In addition to critical reflection on personal and social issues, Vu and Burton (2020) propose that mindfulness encourages moral reflexivity with the potential to transform learning, including management learning in organizations. Perera et al. (2024) suggest that the potential of mindfulness practice to balance cognitive and emotional aspects of decision-making can transform workplaces by promoting more ethical decisions and by mitigating against discrimination. Moreover, in the United Kingdom (UK), a growing number of politicians have started practicing mindfulness and appear to consider mindfulness as more than mental training that brings along individual benefits, instead contributing to a flourishing society (Bristow, 2019). Finally, practitioners call for more rigorous research exploring how mindfulness can help cultivate transformative leadership (Paul, 2023).

However, empirical research examining the potential of mindfulness to transform entire communities is still scarce. In other words, today much prominent mindfulness theory and practice is concerned with cultivating awareness of the self, predominately focusing on the breath to help calm one’s mind and take on the stance of a non-judgmental observer of one’s thoughts and feelings through silent meditative practice (Kabat-Zinn, 2005; Williams and Penman, 2011). Scientific studies focusing on how mindfulness may help transform relationships *between* individuals are more rare than those investigating how it may help cultivate transformation *within* individuals. An exception is case study research of community-based activism in the UK and Germany, proposing that the Buddhist notion of *interbeing* – a term coined by influential Buddhist monk and writer Thich Nhat Hanh which relates to humans being inextricably mutually engaged with each other – is an essential aspect of social change and the transformation of society (Schmid and Taylor Aiken, 2021). Another exception is Tobias Mortlock et al. (2022) mixed-methods study combining individual with collective mindfulness training in a high-stress military setting, suggesting that innovative mindfulness training interventions may cultivate transformative capacity not only for individuals but for entire work teams.

Indeed, scientists report that mindfulness can cultivate beneficial outcomes not only for the self but also for others (c.f. Schindler and Friese, 2021, for a recent review). For example, several studies suggest that brief mindfulness training interventions may be effective in helping workers behave more prosocially (Hafenbrack et al., 2020) and that even 8 to 15 min of mindful breathing can increase workplace civility (Hafenbrack et al., 2024). Other empirical work (by the same lead author) indicate that being in a state of mindfulness may in fact *reduce* people’s motivation to feel guilt or engage in prosocial reparative behaviors (to mend broken relationships; Hafenbrack et al., 2022). While meta-analytic analyses do report that there is a significant correlation between mindfulness practice and prosocial outcomes (Berry et al., 2020; Donald et al., 2019), these comprehensive reviews also highlight concerns about publication bias and challenges regarding replicating these findings. Notably, Berry et al.’s (2020) meta-analysis makes a critical distinction between the cultivation of compassionate and empathetic attitudes through mindfulness practice and the translation of these attitudes into actual prosocial behaviors, particularly when such behaviors would incur a cost to the individual (e.g., sharing expertise with a colleague or offering shelter to a person in need), and conclude that there is no conclusive evidence supporting the universally salubrious effect of mindfulness meditation on actual prosocial behavior. It may be that it matters more than we previously thought *how* people practice mindfulness together for social benefits of mindfulness practice to occur – hence our particular focus on examining mindfulness practice in groups.

In fact, in the contemplative traditions, mindfulness is understood as a socially engaged practice. As mentioned above, one of the core tenets of Eastern mindfulness is the intent to help people realize their *interbeing* nature. According to Hanh (2020), human experiences and the realities we create are all interconnected, and realizing this lays the path toward collectively understanding and overcoming suffering. We are more interdependent than we think: mindfulness in one person - as well as mindlessness - often impacts the level of mindfulness in another. Recall the last time you said or did something mindless to another person; this has likely influenced their capacity to be, to become, or to remain calm and non-judgmental. By the same token, meditation, the core mechanism of generating mindfulness, can be defined as “the practice of concentration, or stopping and looking deeply, in order to realize the truth of interbeing” (Hanh, 2020, p. 88). This means we can make space to cultivate mindfulness within ourselves, as well as cultivate mindfulness ‘in the space between you and me’.

Based on the above-mentioned theoretical frameworks for understanding mindfulness in groups and organizations, as well as a broader understanding of mindfulness and its benefits, the field is well-positioned to better understand collective mindfulness practices and using qualitative, narrative methods to examine the social purposes of mindfulness.

In this study, we explore the experiences of members of an online mindfulness community of practice at a large metropolitan University who have come together to practice mindfulness and gentle mind–body exercises since the beginning of the COVID-19 pandemic, i.e., approximately 4 years to date. In this university setting, online-facilitated mind–body sessions have been offered by experienced mindfulness facilitators three times a week and participants were invited to log on and join the online mindful community at any point.

The study is qualitative in nature. Sixteen semi-structured interviews were conducted with volunteers from the above online mindfulness community of practice, to gain an understanding of their motivation to engage with the community, to explore how they have experienced the mind–body practices, the community, and any outcomes of being a member of this community. Interviews were transcribed and analyzed using inductive thematic analysis (Braun and Clarke, 2006) by two members of the research team (not the mindfulness facilitators), ensuring adequate interrater reliability, comparing and discussing major themes in two iterations.

In the sections that follow, we situate the study in its theoretical rationale, explain study research design and setting in detail, before presenting the results from our exploratory analyses. The paper concludes with a discussion of the study’s implications for theory and practice as well as an outline of the study’s research limitations and opportunities for follow-up research.

## 2 Theoretical background

### 2.1 Theoretical rationale

People can practice mindfulness alone or they can practice mindfulness with other people in a group setting within a community of mindful practice. Our study focuses on the social purpose of mindfulness, in other words its mission to cultivate wellbeing beyond individual transformation which has not been fully explored. Lacking knowledge about how individual and group processes and outcomes of people practicing mindfulness in communities of practice interact is problematic from a theoretical, practice-based and pragmatic perspective. There are at least two potential avenues through which mindfulness may prompt social transformation as an individual or as part of a community of practice: On one hand, there may be social or interpersonal benefits to an individual practicing mindfulness, for example increased prosocial behavior (Hafenbrack et al., 2020). This improved prosociality may come about because individual mindfulness practice not only helps an individual become aware of and regulate their own emotions and behavior, but this self-awareness and self-regulation may *transcend* the self, prompting prosocial attitudes and actions such as empathy and compassion (Vago and Silbersweig, 2012). On the other hand, when people practice mindfulness together, individuals involved in such group-based mindfulness practice may benefit from another’s mindfulness practice. This is because mindfulness practice can be ‘contagious’, in a positive way: it may prompt *interpersonal* mindfulness, defined as self- as well as other-awareness with nonjudgment and nonreactivity (Pratscher et al., 2018). Khoury et al. (2023) speculate on the mechanisms involved in generating these personal benefits through interpersonal mindfulness: prosocial behaviors initiated by a person who practices mindfulness may facilitate awareness and understanding of internal somatic and emotional states, emotion regulation, empathy and mindfulness of another person in their presence. In addition, developing embodied awareness of the self may contribute to a greater understanding of how the minds and bodies of others interact with the self to enhance interpersonal connection and wellbeing.

Mindfulness and mindful movement based mind–body interventions have reliably been shown to be effective in increasing individual wellbeing in a variety of contexts including workplaces, schools and universities (Creswell, 2017; Bartlett et al., 2019; Vonderlin et al., 2020). In the scientific literature, mind–body practices have been defined as those “whose origins lie outside of the Western culture, typically combining muscle-strengthening, balance training, light-intensity aerobic activity, and flexibility in one package” and include a variety of yoga, tai chi, and other physical activities that also consider mental practices such as mindfulness, relaxation, and spirituality (Powell et al., 2018, 1). Mind–body practices emphasize the interconnectedness of the mind, body and heart in order to soothe the parasympathetic nervous system and strengthen polyvagal tone that in turn allows the individual to gently pause before responding and thus regulate emotion and enhance decision-making (ibid.). The exploration of neurobiological mechanisms underpinning the benefits of mindfulness training have identified measurable changes in the brain associated with attention, perspective taking and cognitive flexibility (Hölzel et al., 2011; Tang et al., 2015; Edginton, 2020) including hemispheric synchronicity (Lomas et al., 2014) and structural changes in the insula, a region of the brain that processes body awareness and emotional awareness (Sharp et al., 2018). A robust evidence base has been established for mindfulness as a mind–body intervention for stress reduction and improvements in wellbeing based on the efficacy of guided practices and inquiry (Farb et al., 2015; Pérez-Peña et al., 2022). The inclusion of inquiry within the group, which fosters connection and a sense of shared understanding, combined with mindful awareness, may foster beneficial change (Pérez-Peña et al., 2022).

The growing evidence base on the efficacy of mindfulness has predominantly focused on in-person groups across a range of community, workplace, educational and clinical settings. More recently there has been an interest in online mindfulness-based interventions which have also been shown to be effective in raising wellbeing and reducing employee stress (Spijkerman et al., 2016; Stratton et al., 2017). The success of these online interventions support earlier findings that the inclusion of group-based mindfulness practices and mindful inquiry may be core components that underpin beneficial changes associated with mindfulness training and the creation of a community of practice. There is some research exploring the opportunities and challenges associated with mindful communities of practice, notably their potential to generate care and compassion in work settings (Correia and Strehlow, 2018). *Online* communities of practice have become more prevalent in recent years, especially in the wake of the COVID-19 pandemic. Little is known in the scientific literature about online mindfulness communities of practice, yet we do know that workplaces interested in bringing people together in an online mindful community need to balance potential concerns (perceived lack of personal connection, fear of cyber bullying, and so on) with potential benefits (in particular convenience and flexibility; El Morr et al., 2020). Our study sits at the intersection of three literatures: social functions of individual mindfulness practice, mindfulness practice in a group setting, and online communities of practice.

### 2.2 Relevant theoretical frameworks

#### 2.2.1 Situated learning theory

Social learning is as simple as it is powerful: people learn by watching other people (Bandura, 1977). Situated learning is an educational theory that emphasizes the contextual and relational nature of learning that occurs in adult education (Herrington and Oliver, 2000) and in Communities of Practice (CoPs; Handley et al., 2006). It is based on Vygotsky’s work proposing that humans develop through social interaction (Vygotsky, 1994). Situated learning occurs when individuals collectively make sense of situations, in particular in non-routine contexts such as when people get together outside of their ordinary work convention (Huzzard, 2004). Critical reflection and contextual sense-making are deemed essential ingredients of situated adult learning (Welsh and Dehler, 2013).

#### 2.2.2 Online communities of practice

Social scientists Jean Lave and Etienne Wenger first coined the term “community of practice” in the early 1990s, describing a group of people who share a passion or concern and who come together and interact regularly in order to learn to do it better (Lave, 1991; Wenger, 1998). These communities are characterized by their shared interest, their collective learning and knowledge creation, and their shared practice and identity (Wenger, 1998). CoPs have been shown to be effective in generating knowledge sharing, learning, and professional development (Monaghan, 2010). In particular, community psychosocial wellbeing is cultivated through CoP and community practice interventions (Ohmer and Korr, 2006). In addition, a recent systematic review of public health CoPs suggests that reflective practice, structured problem-solving, and diverse networking may help in generating beneficial outcomes for CoP participants (Barbour et al., 2018).

Online CoPs, also known as electronic networks of practice, are platforms where participants with a shared concern or passion interact to deepen their knowledge, expertise, and social networking capacity (Zhang and Watts, 2008; Gunawardena et al., 2009). Research has shown that online CoPs have various benefits. They can provide opportunities for individuals to engage in ongoing discussions, share personal experiences, and provide emotional support (Prescott et al., 2020). In addition, they may act as therapeutic spaces, offering support and understanding for individuals facing health challenges (Coulson et al., 2017). Finally, online CoPs foster sustained learning and engagement between individuals in particular if they are characterized by trust and interpersonal commitment (Chang et al., 2015).

#### 2.2.3 Online mindfulness programmes

Over the last decade, mindfulness-based interventions (MBIs) have increasingly been offered online. For example, individuals can join time-bound online MBIs delivered via the internet or group videoconferencing technology, such as the 8-week Mindfulness-Based Stress Reduction (MBSR) or Mindfulness-Based Cognitive Therapy (MBCT) training courses (Moulton-Perkins et al., 2022).

Scholars have begun evaluating the effectiveness of these new formats of mindfulness programmes (Spijkerman et al., 2016; Sommers-Spijkerman et al., 2021). Evidence from one of the first narrative syntheses of 10 online MBSR or MBCT programmes indicates that these may be as effective as in-person delivered mindfulness training, yet only three of these demonstrated moderate to high methodological quality (Moulton-Perkins et al., 2022). More recent systematic reviews and meta-analyses indicates that online MBIs can generate modest but significant benefits (Sommers-Spijkerman et al., 2021; Johnson et al., 2024), yet we still know too little about who signs up for and who drops out of online mindfulness programmes, how often individuals should log on or attend to benefit, or who might benefit most.

Understanding drop-out rates for mindfulness programmes is particularly important because we know that in mindfulness, practice really does matter in terms of helping generate beneficial outcomes (Parsons et al., 2017). This argument is supported in a systematic review of 8 RCTs of online MBIs offered during COVID-19; overall, a more beneficial effect could be detected for MBIs with a longer duration as well as for those who offered repeated intervention options (Witarto et al., 2022). However, according to Vargas-Nieto et al.’s (2024) systematic review of digital MBIs for repetitive negative thought, we lack solid data on drop-out rates for online mindfulness (the authors suggest that only four out of the 13 studies included in their review reported adequate completion rates), and drop-out ranges widely, with completion rates ranging from 21 to 85%.

In addition, a recent systematic review of 56 Randomized Controlled Trials (RCT) s of mind–body interventions to manage chronic pain, delivered using technology-enabled channels, found that only two thirds (that is, 38 out of the 56 included studies) provided a recommended ‘dose’ for adherence, i.e., how often to attend, log on, or practice the recommended techniques to experience benefits (Johnson et al., 2024). The authors of that same review explain that only three quarters of included studies (43/56) tracked intervention adherence, ranging from 69 to 92%, yet measuring this is crucial to gauge the effectiveness of online MBIs. These findings echo the findings of Sommers-Spijkerman et al. (2021) comprehensive meta-analysis of 97 online mindfulness RCTs, reporting overall statistically significant to moderate effectiveness in reducing depression, anxiety, and stress, yet stating that less than 25% of these (22 out of the 97 included studies) had defined cut-off rates for adherence, and over 75% (76 out of 97) did not measure drop-outs.

In terms of understanding for whom online mindfulness programmes might be most beneficial, in Witarto et al.’s (2022) systematic review of online MBIs during COVID-19 a sub-group analysis seemed to suggest that older adults may benefit comparatively more than other age groups; the authors speculate that this may be due to older individuals’ greater capacity for engaging in acceptance-based processes. The same effect was not found across the other systematic reviews and meta-analyses we could identify. Furthermore, a recent systematic review of 13 online MBIs specifically focused on university students found small but significant reductions in depression, anxiety, and stress (yet no link to improved wellbeing), which appeared to show comparatively higher effect sizes than MBIs for other adults (Gong et al., 2023). The authors of that review speculate that this may be due to university students being more familiar with technology-based interventions. In a similar vein, Yogeswaran and El Morr’s (2021) systematic review of (two) online mindfulness interventions to improve medical student mental health suggests these may be effective, yet warn that high drop-out rates diminish this potential benefit. Scholars call for more research specifically exploring the community dimensions of group mindfulness practice facilitated online, to counteract low program usage and high drop-out (Ahmad et al., 2018).

#### 2.2.4 The social effects of individual mindfulness practice

We know that mindfulness practice can reduce symptoms of various mental health conditions (Creswell, 2017), as well as enhance mind–body connection (Grasser and Marusak, 2023), improve cognitive functioning (Lodha, 2022) and strengthen physical health (Cardle et al., 2023). We also know that a disposition toward *interpersonal* mindfulness – an interpersonal awareness of moment-by-moment experiences both within oneself and also within another person by paying attention to the other’s verbal and nonverbal communication – is linked to improved interpersonal communication (Pratscher et al., 2019) and improved intercultural communication effectiveness (Khukhlaev et al., 2022). In addition, *social* mindfulness theory is concerned with paying attention to the interests and concerns of others and by engaging in “other-regarding actions that arise from other-regarding motives” (van Doesum et al., 2013). Social mindfulness can reduce social hostility (van Lange and van Doesum, 2015) and arises via empathy and perspective-taking (Gerpott et al., 2020).

The evidence base on this topic appears incomplete, in an important and arguably understudied way: while we agree that it is important to understand the outcomes of mindfulness training and practice, it is also important to deeply understand the process of how individual mindfulness practice may – or may not – engender social effects. In other words, much empirical work to date has focused on the benefits of mindfulness *programs*, not examining the benefits of *membership* in a mindfulness program. This approach may also contribute to resolving why individual mindfulness practice may not always bring along social benefits, as mentioned in our Introduction.

#### 2.2.5 Mindfulness practice in groups

Nowadays there is an abundance of mindfulness Apps and online mindfulness resources available to individuals interested in learning to practice mindfulness, such as the Headspace™ App or the Calm™ App. However, people typically learn mindfulness practices in groups, for example by attending an 8-week group mindfulness-based stress reduction (MBSR) course based on the seminal work of Kabat-Zinn (1982) and Creswell (2017) or through attending an amended group course based on MBSR or one of its evidence-based derivatives. One of these is the 8-week group program mindfulness-based cognitive therapy (MBCT; Segal et al., 2002). The outcomes of these group-based mindfulness training programs has been studied extensively. For example, in a longitudinal and rigorously designed study comparing MBCT with antidepressant treatment, researchers found that MBCT training is as effective as taking antidepressants even 2 years after completing the program (Kuyken et al., 2015). This impressive finding strongly indicates that learning to practice mindfulness in groups over time is effective.

MBSR pioneer Kabat-Zinn (1982) suggested that the group setting in the course plays a pivotal role in promoting mindful interactions – and thus mindfulness – among participants. There is empirical support for this view: Imel et al.’s (2008) examination of 59 MBSR groups found that being in a group while taking part in an MBSR course accounted for 7% of the variability in reducing psychological stress symptoms. The mechanism for this appears to be driven by MBSR instructors using their mindfulness skills to observe and adapt to group dynamics in real-time, aiming to (a) enhance the group’s collective understanding of mindfulness, (b) improve the group’s ability to listen deeply to each participant’s experiences, and (c) encourage individuals to more openly share their experiences (Imel et al., 2008). Indeed, the group setting in mindfulness practice seems to significantly influence participants’ learning experience – which may be positive or negative – depending on the mindfulness facilitator’s skill in using the “group as a vessel on a shared journey” (Cormack et al., 2018, 735).

Specific examples pointing to the potential superiority of group-based mindfulness meditation over solitary meditation includes improved weight management when meditating in a group (Mantzios and Giannou, 2014) and enhanced social cohesion in groups meditating together (Hanley et al., 2022). Furthermore, a recently published meta-analysis indicates that group-based mindfulness-informed therapy is slightly more effective that standard (individual) cognitive behavioral therapy (Ferreira et al., 2022). Mindfulness practice can also help groups function better overall, because it helps group members become aware of their individual reactions to others in nonjudgmental ways (Michalski and Smith, 2023).

However, other direct empirical comparisons of mindfulness practice in groups vs. practicing alone found no differences in effectiveness of group-delivered and individually delivered MBCT for reducing depression and somatic disease (Schroevers et al., 2016) as well as no differential effect of participating in a mindfulness intervention alone vs. as part of a group on improved character or mindfulness skills (Matiz et al., 2018). This means more research is needed to further illuminate the potential benefits of mindfulness practice in group settings.

### 2.3 Study focus

Bringing together the literatures we have discussed above in the context of the present study, the research question (RQ) for our inquiry is, what are the social functions of group-based mindfulness practice in an online mindfulness community of practice created during COVID-19. Furthermore, we explore this RQ in the context of situated learning theory. This is because the theoretical context for the study is collective reflection, learning, and sense-making.

## 3 Materials and methods

### 3.1 Research setting

This study came about in the context of a large metropolitan university (the first and last authors’ institution) offering 30 min drop-in mindfulness practice sessions via an online platform (Zoom) to staff and students over lunchtime, three times a week. The sessions were run by three experienced mindfulness trainers with specific expertise in Mindfulness-Based Stress Reduction (MBSR), alternating mindfulness facilitation so that there was always one trainer facilitating. Participation was free, no prior meditation experience was necessary, and anyone could join a session at any time. The sessions had been created in the wake of the COVID-19 pandemic to support student and staff wellbeing.

Each session followed the same broad structure: the facilitator welcomed the participants and invited them to share briefly how their mind was (or share a reflection that the facilitator initiated) on a voluntary basis (nobody was forced to share); then the facilitator guided the online group through a 10 to 15 min gentle mind–body meditative practice involving gentle relaxation, mindfulness meditation, and/or gentle stretching practice; and the session finished with another inquiry, specifically an invitation to the participants to share how their mind was then, after the practice (or share anything else related to the practice or session). Throughout, the facilitator followed Crane et al. (2015) disciplined improvisation approach to the inquiry, namely seeking (as much as possible) to foster affiliation and intersubjective connection within the group of people present and gently steer communication toward nonjudgmental sharing of universal, embodied experience (as opposed to story-telling or sharing self-criticism).

The study was conducted during the summer of 2022; 2.5 years after the start of offering the drop-in mindfulness sessions at the university. By then, approximately 330 online mindfulness sessions had been run. Approximately 300 individuals had taken part in at least one session. On average between 10 and 20 individuals logged on to a session, and there were approximately 50 individuals who had participated regularly (i.e., at least once a week for several months). Over the several years that the online mindfulness sessions were running by the time the study was conducted, the sessions were reasonably well-known at the university. People joined and dropped out for a variety of reasons; scheduling conflicts contributed to drop-outs, so did changes in work patterns or individuals moving away and thus into other life contexts, as well as varying degrees of prioritizing practicing mindfulness alone vs. as part of this particular group. The individuals who formed part of our empirical study were drawn from the approximately 50 individuals who joined reasonably regularly, and thus were the community of practice for this study.

Our methodology reporting approach follows APA publication recommendations for qualitative empirical research (Levitt et al., 2018).

### 3.2 Research design

The research design for this study follows an interpretivist research paradigm, meaning that we aim to understand human behavior through subjective interpretation (Denzin and Lincoln, 2005). This paradigm shaped our relativist research ontology, assuming that there are multiple realities in life and different people may experience the same event differently, and a critical realist epistemology, which determined our research question by seeking to understand our participants’ interpretations of the world in their context and through their perceptions (Willig, 2013).

#### 3.2.1 Participant recruitment

Following approval to conduct the study from the first and last authors’ university Institutional Review Board (IRB), participants were recruited on a volunteer basis by sending email communication to all individuals who had attended at least five of the lunchtime online mindfulness practice sessions over the course of a month (as outlined above). The total number of participants was 16. We chose this exploratory sample size leaning on Hagaman and Wutich (2017) who suggest that 16 or fewer qualitative interviews are sufficient to uncover common themes when conducting research with generally homogeneous populations and on Saunders and Townsend (2016) who suggest that the norm for sample size in organizational psychology research is between 15 and 60 individuals.

#### 3.2.2 Participant characteristics

The 16 individuals below volunteered to participate in the study, provided informed consent, and were interviewed by two research assistants not affiliated with the online mindfulness sessions. They were between the ages of 20 to 60 years. In Table 1, we outline the demographics we captured for the participants, notably gender, and their roles (student or staff at the university). Out of the participants, 3 were male and 13 were females, which was representative of the participants who attended. The age range was spread relatively widely; 5 participants were in their 20s, four in their 30s, 3 in their 40s and 50s, respectively, and one person was in their 60s. Five students at the university were interviewed, 7 staff members, and 4 individuals who were affiliated but neither staff nor student at the university.

**Table 1:** Demographics of the participants included in the study.

Name (anonymised)	Gender	Age range	Student or staff
Sarah	Female	20s	Student
Ruma	Female	50s	Student
Olivia	Female	40s	Staff
Ava	Female	60s	Staff
Matthew	Male	30s	Staff
Emma	Female	20s	Student
Zoe	Female	30s	Staff
Sriya	Female	50s	Staff
Lauren	Female	50s	n/a
Sophia	Female	20s	n/a
Emily	Female	30s	Staff
Jessica	Female	40s	n/a
Daniel	Male	20s	Student
Dounia	Female	30s	n/a
Hossnara	Female	20s	Student
Noah	Male	40s	Staff

#### 3.2.3 Interview procedure

A semi-structured interview schedule was developed and pilot-tested before conducting interviews with the research participants. The main focus of the questions was to understand the participants’ experience of the online mindfulness sessions. Questions explored how they found out about the sessions; when they started regularly logging on; what their motivation was for joining; how regularly they attended; how they would describe their experience of the sessions and how this experience may have changed over time; whether they stopped joining at some point and what factors might have contributed to that and/or what drove them to re-join the sessions subsequently; what mindfulness meant to them and how they practiced mindfulness; how they experienced the online mindfulness community; and any other feedback participants were willing to share.

Interviews were arranged via email at a convenient time for the participant and conducted online. Having ensured that informed consent was provided, the researcher ensured that the participant understood the purpose of the study and the procedure. Interviews were audio-recorded following verbal consent from participants; these audio-recordings were destroyed upon transcription. Each interview took between 25 and 40 min and was debriefed in accordance with ethical guidelines.

The informational power among the sample of participants appeared satisfactory (Malterud et al., 2016). This was demonstrated by the fact that both interviewers reported no significant additional new insights collected during their last interview and concluded that data saturation seemed to have been reached (Guest et al., 2020).

### 3.3 Analytic approach

Interview transcripts were analyzed using Braun and Clarke’s (2006) Thematic Analysis in several stages to identify, analyze, and report on findings in the data. Two researchers (the first and second author) developed initial codes inductively and individually, first by hand, then by grouping them electronically, and sharing and discussing these in three iterations. After each iteration discussion, the researchers went back to the transcripts to re-code and re-identify major themes and subthemes before sharing their interpretations again, until intercoder reliability was high and identified themes and subthemes were virtually identical across the two researchers (O'Connor and Joffe, 2020).

### 3.4 Reflexivity

Reflexivity in qualitative research is concerned with researchers critically investigating their own beliefs, judgments, and biases which may skew the reporting of results (Jamieson et al., 2023). In line with the principles of subjectivist research paradigms guiding this study, it is important to note that both researchers involved in the data analysis have been immersed in the study in different ways (the first author served as one of the mindfulness session facilitators; the second author was one of the data collection researchers) and therefore bring a degree of researcher bias to the data analysis (Braun and Clarke, 2012). To mitigate this and minimize bias in reporting, the researchers repeatedly engaged in reflection during the analysis process to realign their understanding about the research process and its aim, and in particular how each of them might be influencing this process (Lazard and McAvoy, 2020). Assumptions and expectations about the data were shared in order to disentangle these from the empirical data as much as possible.

## 4 Results

### 4.1 Summary

Four key thematic codes and their respective subthemes were identified in the data analysis: Collective alignment; common humanity; improved mind–body awareness; and unique mutual trust and connection in the online mindfulness community of practice we studied. Overall, our research participants said they found the online drop-in sessions helpful and they appeared to benefit from being a member of the online mindful community of practice. Particularly noteworthy was that not only did the opportunity to engage in group-based mindfulness practice seem to help improve individuals’ mind–body awareness; it also seemed to help foster a unique sense of social connection among the members of the online community of practice.

All four thematic codes and their subcodes are outlined in Table 2. These capture the core findings from our interviews. The first key theme was about the group-based setting for the online mindfulness drop-in sessions. This seemed to provide a helpful social structure for participants’ mindfulness practice – all the more so as many participants juxtaposed this to the felt sense of social isolation that COVID-19 presented. Second, interviewees seemed to benefit particularly from the fact that online sessions participants were invited to share what was on their minds and how they were feeling before and after the mindfulness practice. This appeared to contribute to them feeling less alone on one hand, and to helping them understand their own personal feelings better. Third, mind–body awareness seemed to have improved through regular participation in the online mindfulness sessions, potentially linked to the regular practice of actively sharing insights and feelings in the group. And finally, the sessions appeared to have fostered a unique sense of social connection among members of the mindful community. More specifically, our interviewees suggested that they felt connected to fellow drop-in session participants in unusually deep and precious ways.

**Figure 1:**
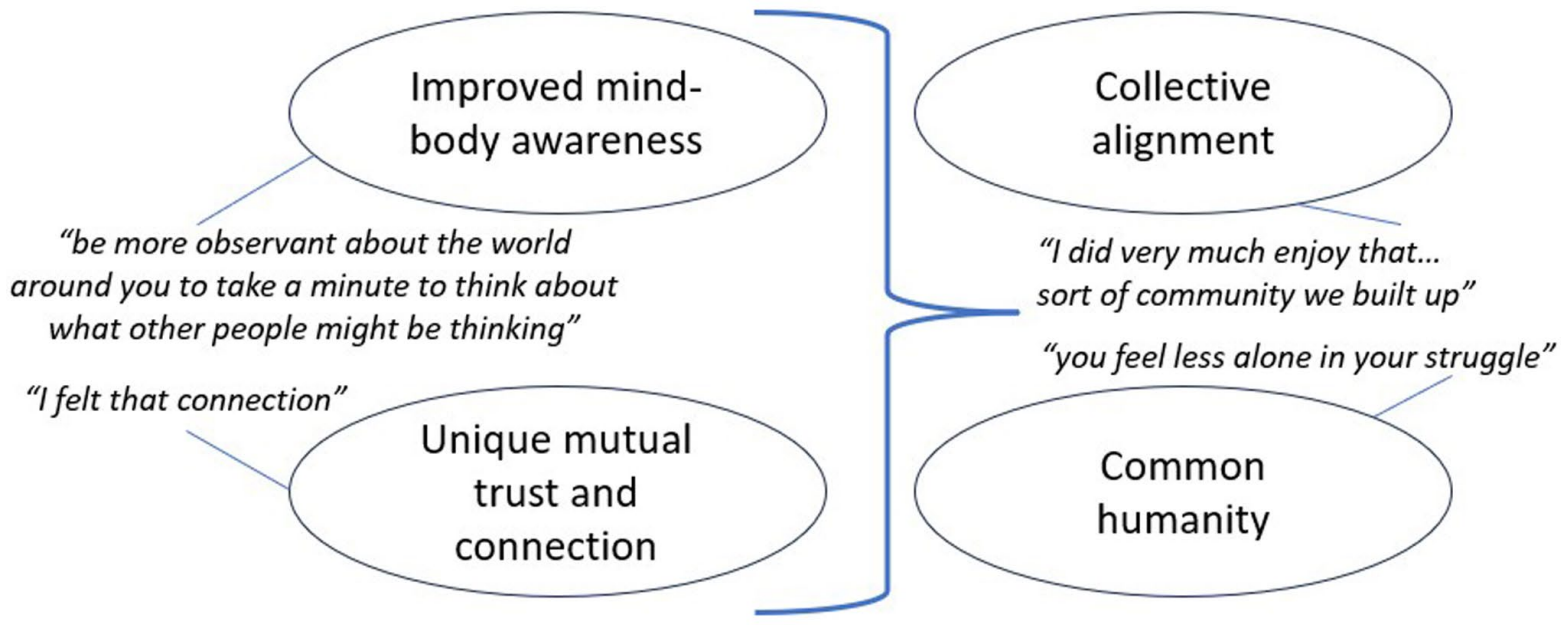
Logical relationship between key themes alongside illustrative quotes.

When analysing the thematic codes further, we put them into two sub-groups, and found that the combination of the first sub-group is likely to have helped bring about the themes in the second sub-group. In other words, improved mid-body awareness *and* unique mutual trust and connection (two of our thematic codes as outlined further below) helped generate a combination of the two other thematic codes; namely collective alignment and common humanity. We therefore arranged the four key themes in a (tentative) logical relationship, as outlined in Figure 1.

Table 2 shows the four key thematic codes used for the data analysis, alongside subthemes and illustrative quotes from interviewees.

**Table 2:** Qualitative themes and subthemes alongside illustrative quotes.

Thematic code	Subtheme	Illustrative quotes
1. Collective alignment	1.1 Helpful structure	*“There was a regular pattern and routine to it” (Sarah)*
	1.2 Help with individual practice	*“It was kind of convenient, and at other times it was necessary” (Ruma)*
	1.3 A sense of community	*“I did very much enjoy that community aspect of it, connecting with the others and that sort of community we built up” (Noah)*
2. Common humanity	2.1 Feeling less alone	*“you were not alone in dealing with the kind of weirdness of situations” (Olivia).*
	2.2 Understanding one’s own feelings better	*“outlet for just like for 20 s saying how I feel and checking in with how I feel” (Ava)*
3. Improved mind–body awareness	3.1 Reconnecting with the body	*“I found myself learning about myself. Basically, I think I had been very detached from myself and my body” (Emma)*
	3.2 Group practice getting them out of the thought bubble	*“[the group] practice helps you to be more observant about the world around you to take a minute to think about what other people might be thinking rather than just trapped inside your own thought bubble” (Daniel)*
4. Unique mutual trust and connection	4.1 Absence of social pressure	*“you just were responsible to be there and to be open” (Zoe)*
	4.2 Mutual care	*“I felt that connection, I felt comfortable talking. I guess that that trust was built and hard for me to pinpoint exactly what lead to that but it did feel like a space where you felt trust and safe” (Sriya)*

Quotes are attributed to interviewed participant by adding pseudonyms per participant.

Each theme is illustrated further below.

### 4.2 Collective alignment

Three sub-themes emerged for this first thematic code; (a) helpful structure; (b) help with individual practice; and (c) a sense of community.

The first of these is concerned with the fact that the online drop-in mindfulness sessions occurring three times a week was perceived as a helpful structure in the lives of the participants. In the words of Noah, *“[I] think it gave a structure to my day … the discipline of attending at a regular time and engaging with the practice, that was really helpful.”* Many of the regular participants in the mindful community had been joining the sessions since the beginning of the pandemic, and the sessions seemed to offer them a regular break from their stressful lives. Several interviewees emphasized that the regular sessions provided much-needed structure for organizing their days. One person suggested *“I remember quite strongly feeling that it was a really nice sort of clearing a space in the middle of your day, which was very good.”* (Olivia). Others said they liked *“there was a regular pattern and routine to it”* (Sarah).

Some interviewees shared that they were somewhat astonished that the short, regular structure of the drop-in sessions proved helpful to them. One regular participant shared that *“expectations were like it’s definitely not going to work, so give it a little trial period, but yeah, pleasantly surprised”* (Ruma). Another reflected on the fact that the sessions were short and in the middle of the work day, adding that she was *“actually surprised what you could get from that”* (Lauren).

In sum, the regularity of the sessions appeared to bring stress relief. The quote below sums up this sentiment:

*“It was very, very difficult in [my work] sector … I’m trying to say that the world I was working in … was … under a lot of strain and devastation really. So it was really … helpful to come to this quiet time for lunch. Usually twice a week, and just to find space to do it.”* (Zoe).

The second sub-theme revolved around the effect of the group-based practice setting: it was perceived as helping the participants with their individual mindfulness practice. Many of the interviewed participants indicated that practicing mindfulness in the online group encouraged them to practice in the first place. As Ruma said, “*it was kind of convenient, and at other times it was necessary*.” Several explained that they found practicing mindfulness with other people easier than practicing alone, saying that *“it would help me with my own discipline of practicing, I find it easier, yes, in a group than to do it myself.”* (Noah). Some of the interviewees had left the online community for a variety of reasons, and insisted that the group setting had been conducive to regular mindfulness practice. The sentiment that the mindful community had been valuable in promoting regular individual mindfulness practice is summarized in the statement below:

*“I am nowhere near as regular with practicing now that I am not practicing online [in the group], and I do not have that outlet for just … 20 s saying how I feel and checking in with how I feel”* (Ava).

The final sub-theme related to the group-based setting of the mindfulness sessions was focused on a sense of community. Specifically, interviewees made statements such as “*I did very much enjoy that community aspects of it, connecting with the others and that sort of community we built up”* (Noah), indicating that over time, the online drop-in sessions had fostered a sense of connectedness and shared experience. In addition, this emerging sense of community seemed to have been perceived as affirming to the participants, particularly by promoting a shared sense of understanding the world around them. Essentially, the online mindfulness sessions provided space for much-needed shared experiences, as expressed in the quote below:

*“It was really validating because during that time there was a collective experience that you were not aware of what was happening until you came into the mindfulness sessions and people were saying, oh I also feel like that and that bit on the news made me feel as well like that and that was very validating.”* (Ava).

### 4.3 Common humanity

Two subthemes are discussed in the context of this second thematic code: (a) feeling less alone; and (b) understanding one’s own feelings better. Both are situated in the context of the invitation by the facilitators to actively share a thought or feeling at the beginning and end of the online drop-in sessions. Interviewees seemed to particularly enjoy sharing at the end of the session and listening to others’ reflections. One person explained, *“coming back to [the practice] and reflecting and what went right and seeing how other people felt it’s good”* (Sophia).

The first subtheme here is about feeling less alone and isolated. Some of this seemed to be specifically because of listening to other participants share some of their struggles. In the words of one interviewee, actively sharing during the practice meant *“hearing the types of issues that other people are struggling with, so that you feel less alone in your struggle”* (Daniel). Another person related that sharing how they were feeling *“was very useful because you just saw that you have a bigger whole, you know, you were not alone in dealing with the kind of weirdness of situations”* (Olivia).

The second subtheme relates to understanding one’s own feelings better, because of being in a context in which individuals are encouraged to actively share their thoughts and feelings. Being gently encouraged to share what was on their minds seemed to provide an opportunity to work out in the first place what *was* on their minds, in that moment. In the words of one interview participant, the online community offered an *“outlet for just like for 20 s saying how I feel and checking in with how I feel”* (Ava).

Expressing feelings was deemed superior than silent meditative practice alone. This is because the act of articulating out loud how participants were feeling was seen not only as an opportunity for connection but also an opportunity to understand more deeply what was real for the person in that moment. The quote below illustrates this insight:

*“We can write things down, we can notice ourselves, but when we articulate it to a group and possibly get some, some feedback, or sometimes some support … and actually hearing yourself speak out the words… it’s different from just thinking … I’m acknowledging more deeply how I’m feeling when I say it aloud to somebody else”* (Sarah).

Moreover, listening to others reflect on their mental state during the mindfulness practice was deemed valuable, precisely because other people’s insights seemed to help generate personal insight. In the words of one of our interview participants:

*“It was great to, in the group, connect with people in different situations from myself, because sometimes that helps, helps with reflection to understand that everyone’s circumstances and my own trends is transient. They’re not fixed.”* (Matthew).

### 4.4 Improved mind–body awareness

The third key thematic code refers to improved mind–body awareness. This is in itself not surprising as mindfulness practice generally fosters mind–body awareness. Yet this increased mind–body awareness seems to also have come about because the group setting in the online mindfulness community seemed to have enabled learning about the self.

As expected, about half of the interviewed participants in the online community identified that the mindfulness drop-ins had helped them improve their mind–body awareness, in other words their embodied felt sense of being present in mind and body. Facilitated mindfulness practices included gentle yoga stretches, exploring different types of perceptual awareness such as focusing and subsequently broadening attention on particular aspects of seeing, listening, feeling and so on, as well as mindful breathing and mindful movement. The two sub-themes here were (a) reconnecting with the body; and (b) the group practice getting them out of the thought bubble.

First, several interviewees mentioned that they welcomed the regular opportunity to consciously shift attention onto themselves. An opportunity they would not ordinarily use even if they blocked time in their diaries to *“have 20 min quiet time … I do not think I would have engaged with myself quite as much as they allowed me to engage”* (Emily).

The core insight here is that their awareness of their five senses seemed to have improved. This, in turn, seemed to have strengthened their sense of connection between mind and body. The idea of *re*connecting mind and body was central to this theme, with an interviewee recalling the following:

*“I found myself learning about myself. Basically, I think I had been very detached from myself and my body. For most of my life, and I think practices like mindfulness has really helped me to connect”* (Emma).

Second, and perhaps more interestingly, the mindfulness practices seemed to have provided a welcome break from being lost in thought and reconnecting with others and with the world around them. Becoming more embodied seemed to be at the heart of this theme, with interviewed participants explaining that they enjoyed getting out of their minds and getting back into consciously feeling their body alongside others. The notion that the practice *“relaxes your body and relaxes your mind”* (Emily) was a common theme here among interviewees. One participant reflected on the positive energy that could be felt between individuals getting together to practice mindfulness, adding that *“if you have got a whole room full of people meditating and feeling calm, there’s something that’s happening on a subconscious cellular level that adds to the experience”* (Daniel). This effect is particularly noteworthy as people were not physically in the same room yet a different, beneficial energy seemed to emerge nonetheless. The same participant summarized this benefit of practicing together, online, as:

*“[the group] practice helps you to be more observant about the world around you to take a minute to think about what other people might be thinking rather than just trapped inside your own thought bubble”* (Daniel).

### 4.5 Unique mutual trust and connection

The final key thematic code is unique mutual trust and connection. The following two subthemes emerged on the impact of the drop-in sessions for the interviewed participants and point toward a unique degree of trust and connection that some of the participants appeared to have felt toward each other. They are (a) absence of social pressure; and (b) mutual care. Both of these relate to the fact that people from a wide range of groups were invited to participate in the drop-in sessions, including current and former students and staff members. Several interviewees commented on the fact that different people from different parts of the organization would be *“coming together to reflect and think and take this time out,”* and added *“I think [connecting with really different people] is a really powerful thing”* (Olivia).

The first of these subthemes is about the somewhat paradoxical idea that this particular social setting did not bring with it the usual social pressure to follow conventional norms of behavior, such as being nice or outwardly taking care of each other. Participants expressed in particular a sense of relief that the sessions were not about being *“responsible to look after people”* and at the same time they welcomed the fact that *“you just were responsible to be there and to be open”* (Zoe). In other words, whenever someone logged on to a particular mindfulness drop-in session, they would not need to behave in a particular way toward each other and instead were allowed to simply *be*.

Notably, it seems that being released from this particular social pressure meant that session participants could be genuinely there for each other, *“listen to each other and respect each other and also give each other space” (Zoe).* The lack of social pressure in this setting was mentioned by several interview participants as valuable, precious even, as the statement below suggests:

*“I just felt I did not feel any pressure to be a certain way or hold feelings for anyone or if I was feeling really stressed, anxious, or down I could just come with that to the mindfulness without having to pretend that it wasn’t there or be a certain way. Yeah, that was a really unique space that was completely different to being with friends and family”* (Ava).

The second subtheme in this category leads on from the first, in that interview participants shared that there seemed to be mutual care among session participants as a result of the unique social bond that people felt for each other. An interviewee explained that in the sessions *“there’s a sense of nurturing, so it feels very psychologically safe, of caring about ourselves and each other”* and that *“people have mentioned things that they are struggling with, or ways that they were feeling that were fairly personal and intimate, in some cases, you know, and what they got back from the group was support and loving kindness”* (Daniel). Essentially, the community seemed to offer a space for giving and receiving social support informally.

This sense seemed particularly palpable among participants who joined the sessions frequently. In essence, the more frequently people participated, the stronger this sense of mutual care seemed to become, which meant that *“the people who were joining regularly were very willing to be vulnerable, to share how they are, which I have never experienced before”* (Jessica). In the words of another one of our interviewees:

*“I felt that connection, I felt comfortable talking. I guess that that trust was built and hard for me to pinpoint exactly what lead to that but it did feel like a space where you felt trust and safe”* (Sriya).

One participant, however, indicated that the online nature of the group meant that the connection was less natural than it would have been in a face to face setting. She explained, *“there was less of a human connection with the others, we had a bit of a chat, and I could relate to some of what they were saying but there was less room for that side of things which I would have liked”* (Lauren). Clearly, online connection cannot really replace real human interaction and connection.

In sum, the data indicates that an atmosphere of mutual trust and care seemed to have emerged for the majority of the people interviewed for the study *despite* an absence of pressure to act in conventional ways toward each other.

We discuss these findings and what they may contribute to theory and practice in the section below.

## 5 Discussion

This inquiry is about exploring the social functions of group-based mindfulness practices in an online mindfulness community of practice created during COVID-19, with a particular focus on understanding the process – and potential benefits – of being a member of a community of practice engaging in regular gentle mind–body exercises together over Zoom. We examined the exploratory qualitative data we collected within a situated learning context. In other words, the underlying assumption for our investigation was that the members of the community of practice under study would engage in learning in the specific situation in which their learning occurred.

Besides drawing on situated learning as context, we structure the discussion through the lens of interdependence theory, a framework that examines the influence of social orientations, such as cooperation or conflict, within contexts where outcomes are interdependent (Kelley and Thibaut, 1978). This is for the following reasons: While we acknowledge that mindfulness theory and practice needs to understand intrapersonal (or intrapsychic) processes, it is helpful to make sense of our findings with an interdependence lens. Interdependence theory asserts that it is the interpersonal dynamics that predominately shape individuals’ perceptions, motivations, and behaviors (Rusbult and Van Lange, 2008). Essentially, the theory posits that these interpersonal interactions form the emotional landscape within which individuals make decisions and take actions. In addition, interdependence theory offers a fruitful pathway to integrate mindfulness theorizing with the contemplative tradition’s emphasis on other-orientation and interdependence, aspects that may not yet have been fully explored in the contemporary mindfulness discourse (see Gergen, 2009). Echoing the Dalai Lama’s insights, profound wisdom is realized when individuals acknowledge and value the interconnectedness of their own interests with those of others (Dalai Lama, 2005).

### 5.1 A special note on the special context of this study

Before outlining the study’s proposed contributions to theory and practice, it is necessary to draw attention to the fact that the study was conducted during the COVID-19 pandemic, and every reader will know that this was an unprecedented time of apprehension and ambiguity for most. It is reasonable to assume that mindfulness practice is well-suited to address feelings of uncertainty, loss, and confusion that inevitably came along with the pandemic (Antonova et al., 2021). There is also evidence that mindfulness appears to have been protective against negative affect arising during COVID-19 (Treves et al., 2023). Moreover, in a systematic review of 16 nonpharmacological interventions developed during the pandemic to promote the mental health of children that include mindfulness, Quiroga et al. (2025) found that these were potentially effective. The authors also suggest that interventions designed during COVID-19 are likely to be useful in other future crisis situations, yet note a significant risk of bias across the studies they examined, hence caution against drawing firm conclusions.

Our study is no different in this regard: it was conducted during an especially unusual time, its design prevents us from making any generalizable predictions, and it is situated in a scientific literature that is still in its infancy. As a case in point, online group psychotherapy pioneer Haim Weinberg who had been facilitating online discussions on the topic among 400 group therapists from 30 nations for over 25 years synthesized these insights in his (2020) practice review of online group psychotherapy for the COVID-19 context. His recommendations included that the lack of physical presence in virtual meetings and distorted eye contact may warrant increasing therapists’ self-disclosure (TSD) and enhanced verbal interactions. While there is certainly scientific support for the use of TSD in therapeutic settings, a more recently published study of two independent samples of therapists (*N* = 1705) and patients (*N* = 772) interacting online early on during the pandemic suggests that therapists perceive the use of TSD as more helpful in fostering real relationships than patients (Luo et al., 2024). Clearly, the COVID-19 pandemic helped accelerate our understanding of online group therapeutic interventions, including online mindfulness groups. Yet scholars call for more research to better grasp their potential (Andrews et al., 2024; Quiroga et al., 2025). Our study responds to this call.

### 5.2 Implications for theory

**Figure 2:**
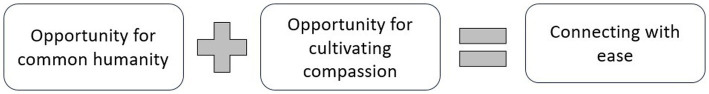
Logical relationship of propositions for how to create a mindful community of practice.

Based on our empirical investigation, we make three exploratory propositions intent on stimulating follow-up empirical research at the intersection of literatures on online communities of practice, mindfulness practice in groups, and the social effects of individual mindfulness practice. We have arranged these exploratory propositions in a logical relationship, as depicted in Figure 2. In essence, we speculate based on our exploratory data set that the combination of proposition 1 and 2 may result in proposition 3, and all three may contribute to creating a mindful community of practice.

Taken together, these propositions aim to stimulate further empirical research in this understudied area, by formulating a proposed – and testable – combination of elements for how to create a mindful community of practice. Leading on from the sections outlining this work’s implications for theory and practice below, we outline follow-up research opportunities for further empirical examination, potential correction, and extension of our propositions.

#### 5.2.1 Proposition 1: creating opportunity for common humanity

Our data suggests that the online mindful community of practice we studied first and foremost helped individuals experience common humanity, in other words, share a felt sense of belonging, at least during the time they practiced mindfulness together. The participants we interviewed repeatedly mentioned that the online mindfulness community provided respite from the isolation many people felt because of the COVID-19 pandemic.

A basic assumption in mindfulness is that there is suffering in the world, and this suffering can be alleviated through mindfulness practice. In the context of this study our data indicates that the mindfulness-based community of practice we examined helped individuals enjoy a sense of community, even if only temporarily.

This is because in our study, the personal mindfulness practice that was cultivated because individuals regularly logged on to the online mindfulness group seemed to help them feel less alone (thematic code 2.1), understand their own feelings better (thematic code 2.2), and the group practice seemed to get them out of their own thought bubble (thematic code 3.2). Thus they appeared to become better able to recognize helpful as well as unhelpful thoughts, emotions and impulses with a deeper awareness of universal experiences, challenges and concerns leading to authentic connection and a sense of belonging within the community of practice. Mindfulness scientists have been able to reliably establish the two-fold mechanism through which mindfulness practice operates; consciously experiencing awareness as well as acceptance is key here (see Carmody et al., 2009; Vago and Silbersweig, 2012; Lindsay and Creswell, 2017). The group setting appears to have served as a facilitator for this, because our participants indicated that their individual mindfulness practice improved in the group setting. This echoes the writings of Thich Nhat Hanh who emphasized that the practice of mindfulness should be a socially engaged practice rather than something individuals cultivate in isolation of others (Hạnh, 1998).

From an interdependence perspective, experiencing common humanity also involves reducing the power of *ego*. In the mediative traditions *ego* is explained as a sense that the self exists entirely independently and separately from others, which leads to ignorance, paranoia, and confusion (Trungpa, 2002). The mind–body practices intent on fostering stronger embodiment in our community participants seemed to have helped them to relax into their bodies, and appreciate their common humanity, which appeared to have offered some respite from being lost in their “thought bubbles” and sense of existing as separate from others. According to mindfulness philosophy this helps individuals realize that they “no longer have to maintain the existence of ego [and] can afford to be open and generous” (ibid., p. 168). We speculate that the regular, repeated group setting of the community of practice may cultivate this stance of openness comparatively more than when individuals practice mindfulness by themselves.

This is why we propose the following:

*Proposition 1: Practicing mindfulness in a community of practice may help create opportunities for experiencing common humanity.*

#### 5.2.2 Proposition 2: creating opportunity for cultivating compassion

Leading on from Proposition 1, the mindful community of practice we studied appeared to have created opportunities for cultivating compassion among its members. Compassion has been defined as a distinct emotion geared at facilitating cooperation and an intent to protect those who suffer (Goetz et al., 2010). Interdependence theory posits that people think and act in relation to each other. A growing body of mindfulness scholarship is focusing on the mental space between individuals, arguing that *interpersonal* mindfulness – the state of being mindful while interacting with others – helps shape healthy relationships (Pratscher et al., 2019). Interpersonal mindfulness practices and trainings based on Gregory Kramer’s Insight Dialog (Kramer, 2007) such as relational mindfulness (Donaldson-Feilder et al., 2019; Donaldson-Feilder et al., 2022) have become increasingly popular in mindfulness science and practice, because of their growing evidence base in fostering *interpersonal* awareness and acceptance.

In particular the combination of feeling a part of a community (thematic code 1.3) and mutual care (thematic code 4.2), coupled with an absence of social pressure (thematic code 4.1) seem to have produced this effect. As the data in this study suggest, research participants indicate that by listening to each other during the online drop-in mindfulness sessions, they experienced a sense of community that seemed unique and precious in its warm and supportive quality. This is related to how compassion is defined in the contemplative traditions. Compassion is basic warmth toward oneself and toward others, which can be operationally defined as an absence of interpersonal aggression (Trungpa, 2002). This warmth is crucial for the development of healthy relationships.

We speculate that in the online mindful community of practice we studied, the foundation for compassion may have been cultivated. We suggest this because communication in the online mindfulness community of practice was carefully managed by the facilitator. Specifically, the facilitator encouraged a ritual of listening to what others were sharing at the beginning and end of the online mindfulness practice sessions. The act of listening to each other at the beginning and end of the mindfulness sessions seemed to have enabled individuals to engage in socially induced processes of *decentering*; shifting their perspective to gain psychological distance (Bernstein et al., 2015; Shapiro et al., 2006). Decentering, also referred to as *reperceiving*, is typically discussed in the context of intrapsychic experiences, in other words, the metacognitive practice of shifting one’s perspective “from *within* one’s subjective experience *onto* that experience” (Bernstein et al., 2015; p. 599, emphasis added). In the social context we discuss here, decentering may have played a role in community building, because it may have fostered a mental shift for the members of the mindful community of practice, from an exclusive focus on *personal* wellbeing through mindfulness toward *interpersonal* wellbeing. This is similar to how Epstein (2013) conceptualizes the link between mindfulness and psychotherapy, essentially suggesting that listening to others enables a shift in mindfulness practice from a solitary and self-focused aspiration to watch one’s own thoughts and feelings toward an interpersonal meditation that helps cultivate compassion between people.

The repeated nature of this interpersonal communication ritual may have been the second ‘ingredient’ for how to create a mindful community of practice. This is why we propose the following:

*Proposition 2: Practicing mindfulness in a community of practice may help cultivate compassion.*

#### 5.2.3 Proposition 3: connecting with ease

Mindfulness is multifaceted (Daniel et al., 2022) and multi-dimensional (Sutcliffe et al., 2016). This means we can practice mindfulness to make space *within* ourselves, and we can also focus our attention mindfully on the space *between* people. More specifically, our data overlaps with Vogus et al. (2014) who theorized that the affective (or mood-based) foundation of a mindful group are equanimity and a prosocial orientation; in other words when people interact with each other with motivations marked by equanimity and prosociality, collective mindfulness emerges (Vogus et al., 2014). We speculate that the particular, unique type of social connection marked by mutual trust and connection that our participants have described (theme 4) is linked to increased prosociality and enhanced equanimity. Additionally, equanimity may be related to our data’s themes of understanding one’s own feelings better (theme 2.2) and in particular the group practice getting them out of the thought bubble (theme 3.2).

This paper is about creating a community of practice, of a particular kind: a *mindful* community of practice. In Buddhism, the essential pillars of mindfulness practice are referred to as the ‘three jewels’: the teacher or facilitator (in Buddhism this has originally been the Buddha); the teaching elements or topics to focus on during the practice (traditionally referred to as the *dharma*); and the community of mindfulness practitioners (referred to as the *sangha*; Hanh, 2020). Of course, in a traditional Eastern contemplative context, the *sangha* would consist of monastics coming together to meditate, but in today’s world this word also refers to a community of Buddhist practitioners regularly practicing mindfulness together. While this paper is not concerned with religious or spiritual mindfulness practice, we argue that creating connections among mindfulness practitioners during mindfulness practice may be an important element of mindfulness, perhaps not emphasized enough in the scientific community studying mindfulness meditation over the last four decades.

People who interact with each other mindfully seem to have one *collective mind* (Weick and Roberts, 1993). A visual metaphor for this is a flock of geese flying through the sky in unison, with each goose adapting its individual flight path to align with the direction – and needs – of the flock as a whole. Interdependence theory conceptually overlaps with Hanh’s notion of *interbeing*, because both emphasize the inextricable connection between people that shapes people’s lives and their experience. Connecting with each other has been at the heart of the community of practice we studied. Especially the sense of relief that participants shared about feeling an absence of the typical social pressures that many of us experience in conventional social settings, such as making small talk, comparing oneself to others, and so on (theme 4.1) seemed to have cultivated what we call *connecting with ease*.

Experiencing ease and thus an absence of pressure is an essential aim in mindfulness practice. The word “budh” in Buddhism means “to wake up,” “to understand at a deep level.” As referred to at the outset of this paper, the purpose of mindfulness is to understand and transform suffering (Bodhi, 2011). Therefore, helping individuals ‘wake up’ from suffering and the potential fear of interpersonal connection is an essential component of creating a mindful community of practice. Today, many individuals in industrialized nations suffer from loneliness and social isolation, shying away from forging meaningful social connections, which in turn puts them at risk for premature mortality (Holt-Lunstad et al., 2015). Among our participants, there was a felt sense of delight in connecting with others, coupled for some with a certain degree of surprise at experiencing a lack of social pressure in this setting. We speculate that many of us in today’s world may benefit from experiencing anew that social connection can be healing and that it can reduce, rather than increase, pressure and stress.

We therefore suggest that to help transform suffering for oneself as well as for others, which is at the core of the intent or purpose of mindfulness practice, it may be helpful to foster connections among mindfulness practitioners with an emphasis on ‘waking up’ from the struggles we all face in our lives by connecting with each other regularly, as Buddhist meditators have done in a *sangha*, in ways marked by equanimity and prosociality. Leading on from this, we propose the following:

*Proposition 3: Practicing mindfulness in a community of practice may help facilitate connecting with ease.*

### 5.3 Implications for practice

Clearly, group mindfulness practice requires skillful facilitation. The competence of mindfulness facilitation can be learned through a variety of reputable mindfulness training institutions globally, and is typically assessed through the evidence-based Mindfulness-Based Interventions Teaching Assessment Criteria (MBI; TAC; Crane et al., 2013). In addition, the characteristics of inquiry in group-based mindfulness practice can be likened to “disciplined improvisation”; flexibly interacting with participants after mindfulness practice in ways that build intersubjective connection and interpersonal affiliation (Crane et al., 2015).

To the best of our knowledge, there is a lacuna of academic research on how to create a mindfulness-based community of practice. Leaning on Wenger (1998) and Lave (1991) who suggest that communities of practice need to consistently foster a shared sense of interest – here mindfulness – as well as a felt sense of community and regular practice of the shared interests, we therefore make the following specific recommendations for individuals intent on creating a mindful community of practice, organized around three main themes. This may be especially important during periods of societal change – and today’s world seems to be marked by ongoing social change, as well as heightened anxiety and uncertainty.

#### 5.3.1 Facilitate regular and varied mindfulness practices

Make it easy for people to join in regular group mindfulness sessions. Offer short sessions at several different times and days a week. Online mindfulness practice is becoming increasingly common and is convenient for people to log on to.

Include gentle yoga, mindful movement, and other mind–body explorations in the mindful community practices, to strengthen the conscious link between mind and body among group participants.

Explore different ways in which community members may experience mindfulness in the group practices. According to Crane et al. (2017), mindfulness-based training always needs to include essential elements such as an understanding of human suffering and mental health – and depending on the needs of those practicing mindfulness, new and different elements may be added, such as varying the degree of physical activity during the mindfulness session. Experiment by introducing community members to different practices and inquire which ones may be more appropriate for the community of practice.

#### 5.3.2 Facilitate connection with ease

Ensure that all mindfulness practices are participant-centered and grounded in mind–body awareness as well as non-judgmental interpersonal sharing. If appropriate, then gently encourage people to build personal relationships in informal ways.

Refrain from managing group membership or attendance. Keep participation voluntary and open.

#### 5.3.3 Facilitate compassionate communication

Invite participants in a group mindfulness session to share what is real for them, without forcing participation from anyone. Lead by sharing authentically yourself. If appropriate, you may want to engage in *leading with vulnerability*, in other words, sharing what you feel in the moment, rather than saying what you may believe others want to hear.

Consider integrating the offering of a mindful community of practice with other workplace initiatives such as training and development, induction activities, or during regular organizational meetings. This may increase the potential of embedding the routine of people coming together to practice mindfulness regularly.

### 5.4 Limitations and follow-up research opportunities

As noted previously, this study was conducted during an extraordinary time, with a group of participants who came together during Covid-19. A small group of volunteers from the mindfulness community of practice were sampled, which means that insights captured were bound to be biased toward those of research participants, rather than expressing more universally applicable views. It is plausible that participants in the sample shared a subset of relevant insights, or other insights were not represented in the data. Furthermore, the interview questions were exploratory in nature, and the lack of targeted questions and our exploratory analysis made it impossible to test whether the online mindfulness community of practice was beneficial, and how. Of course, the fact that only one mindfulness community of practice was sampled further restricts the potential to generalize from the findings presented here. In addition, while the interviewers collecting the data for this study were not members of the community of practice, it is conceivable that participants did not freely share all feedback, as it was known to them that at least one of the facilitators of the sessions was involved in the research study. Finally, there is also risk of bias because two of the authors of this study were involved in delivering the online mindfulness sessions, and one of the interviewers was involved in the data analysis.

Follow-up research can extend the insights presented here in several ways. First, it would be helpful for future research to test out the suggested propositions on how to create a mindful community of practice, for example by exploring the relative contribution of individually focused mindfulness practices versus interpersonal elements in the community. Second, quantitative surveys of mindfulness groups could investigate the attitudes of participants toward their own wellbeing, their learning, and the relationship quality with other participants. Constructs such individual mindfulness, team mindfulness, and psychological safety could be included in measures in such studies, to understand the relationship between individual-level outcomes and interpersonal outcomes. Finally, more longitudinal explorations of mindfulness groups would help us understand the characteristics of how a mindful community of practice is formed and sustained.

## 6 Conclusion

This study took place during COVID-19, a highly exceptional period in the life of everyone. Its specific aim was to explore the social functions of group-based mindfulness practice facilitated regularly online at a large metropolitan University during that time. Findings suggest overall that the online mindful community may have offered a welcome and unexpected safe space to cultivate mutual trust and connection, as well as increased mind–body awareness. These two key factors seemed to be linked to a sense of collective alignment and common humanity. Our findings are discussed through an interdependence theory lens and result in three exploratory, testable propositions on how to create a mindful community of practice.

While the study focused on a mindful community of practice that was formed during a time of unprecedented instability and extreme social isolation for many, and while its research design and exploratory analysis render it impossible to draw firm conclusions, it nonetheless sheds new light on how mindful pillars of interbeing and connection may be formed in an online community of practice. We argue that more research is needed in this understudied domain, in order to extend the transformative potential of mindfulness for one and all.

## Data Availability

The raw data supporting the conclusions of this article will be made available by the authors upon request, without undue reservation.
